# Achieving cross provincial comparisons of osteoporosis screening performance from administrative health data.

**DOI:** 10.23889/ijpds.v4i1.1116

**Published:** 2019-12-05

**Authors:** JK Kueper, MW Alsabbagh, S Peterson, ST Wong

**Affiliations:** 1 Department of Epidemiology and Biostatistics, The University of Western Ontario, Kresge Building, 1151 Richmond Street, London, Ontario, Canada, N6A 5C1; 2 School of Pharmacy, University of Waterloo, 10A Victoria St S, Kitchener, Ontario, Canada, N2G 1C5; 3 Centre for Health Services and Policy Research, University of British Columbia, 201-2206 East Mall, Vancouver, British Columbia, Canada, V6T 1Z3; 4 School of Nursing and Centre for Health Services and Policy Research, University of British Columbia, 201-2206 East Mall, Vancouver, British Columbia, Canada, V6T 1Z3

## Abstract

Administrative databases can be used to measure healthcare performance. This can lead to identification of high-performing practice characteristics and inform innovations. However, a key challenge is that administrative data cannot be easily combined across provinces. Comparable measures must be defined across provinces but operationalized within each province. The purpose of this work is to provide an example of defining a population health concept, osteoporosis screening, and creating measures to examine it across British Columbia, Ontario, and Nova Scotia, Canada.

TRANSFORMATION is a study that seeks to improve the science and reporting of Primary Health Care performance. We used administrative data from the above three provinces to examine osteoporosis screening in those aged 65 years and older. Challenges of databases with different data elements and levels of methods development (e.g. macros) can be overcome for purposes of cross-provincial comparisons. Flexibility of analytic methods and frequent communication is needed.

## Introduction

The ability to compare healthcare performance across jurisdictions using administrative data is not widely understood. We describe our process from developing a meaningful and comparable definition of osteoporosis screening to operationalization across British Columbia (BC), Ontario (ON) and Nova Scotia (NS) provincial datasets. Researchers may adopt a similar approach to derive comparable indicators for osteoporosis screening in other geographical regions or for other aspects of healthcare performance.

## Measure Identification and Definition

Literature searches for Primary Health Care performance measures that could be operationalized using administrative data identified osteoporosis screening, which is a key measure of population health and may be ordered by primary care (most commonly) or specialist physicians [1–5]. To facilitate communication between analysts and other study members, a working definition of the measure (beginning with the wording in the literature) was maintained in a shared document on Huddle—a secure, cloud-based platform designed for project collaboration [6]. ***Final definition:*** Percentage of people who turn 65 years old in the first year (fiscal year (FY) 2013-14) of a three-year study period who have a bone mineral density (BMD) test within the study period (FY 2013-14 to FY 2015-16). Higher percentages indicate better performance.

Knowlton and colleagues’ (2017) framework for generating quality, comparable data from multiple sources was used to arrive at the final definition [7]. A chart was shared whereby analysts recorded potential datasets and variables for creating the measure in their respective provinces. Weekly phone meetings allowed discussion about feasibility and modifications to the working definition. Frequent communication was necessary to evaluate whether comparable measurement could be derived from independent provincial datasets—this required knowledge of the data and healthcare context in each province, as well as an understanding of minimum content criteria for the measure to maintain validity (i.e. how much data manipulation can happen before it loses meaning). Two provinces created the measure; details are outlined below.

## Data Sources

Osteoporosis screening is identified through provincial physician billings for BMD testing. Since each province is responsible for the delivery and organization of healthcare, each province maintains a separate billing system and thus independent databases, each with different dates of availability and levels of data granularity. Ontario categorizes BMD testing according to the number of screening sites, risk level, and whether it is a baseline or follow-up test. British Columbia distinguishes between whole and partial body testing. For part of the study period, BMD tests in NS were billed by the interpreting physicians (radiologists) as “bulk billing”—a group of tests that are associated with a number of patients without the ability to uniquely identify individuals. Thus, a comparable, valid measure could not be derived using the NS data available at the time.

## Operationalization

Analysts calculated the measure separately for their province. Provincial analysts have the best understanding of their respective data sources and share insights that may be missed if a single person performed all analyses. Communication and documentation are crucial to recording decisions and ensure standardized procedures such as variable selection. Comparability needed to be established for the measure’s timeframe, denominator, and numerator.

### Timeframe

Ontario had more data available than BC; both provinces were restricted to a common timeframe of FY 2013-14 to 2015-16.

### Denominator

The overall cohort of interest included people who turned 65 years old in FY 2013-14 and were alive the entire timeframe. The age criterion was based on Canadian BMD testing guidelines [2]. We only included those turning 65 since people 65 or older at the start of the 3-year period could have had a test outside our available data. People with a screening test prior to the study period—if such data were available—would be excluded from both the numerator and denominator. Final eligibility criteria:

*Inclusions:*

1. Turned 65 years old from April 1, 2013 to March 31, 2014.
2. Registered with the provincial health registry for at least 75% of each year (274+ days/year).

*Exclusions:*

1. Records with invalid health card number, invalid or missing date of birth, and invalid or unknown/missing sex.
2. Cessation of provincial health coverage due to death or moving out of the province before the end of the study.

### Numerator

The subset of the denominator cohort who received at least one BMD test in FY 2013-14 to 2015-16. The BMD tests are identified using fee for service items: codes that document each service provided by a practitioner. The ON Schedule of Medical Benefits and BC Medical Services Commission Payment Schedule list each fee code with the amount paid to the practitioner for the service provided. We include all fee codes relevant for our analysis time frame (Table 1); fee codes can change over time. Several codes capture BMD and there is a trade-off between reducing validity of a measure for within-province assessment and increasing validity for between-province comparison. To create a comparable measure, both BC and ON sacrificed some level of detail by collapsing codes together, ON more-so. For example, ON codes differentiate high- and low-risk patients; high-risk patients may have an existing osteoporosis diagnosis and would ideally be excluded. However, since these high-risk patients could not be identified through BC codes, ON included them to maximize comparability. Despite some loss of within-province detail, our data definitions across provinces provide enough information to assess how well evidence-based recommendations for osteoporosis screening are being met. A dichotomous variable for ‘screening test present’ was created whereby people in the denominator cohort with at least one physician billing code for a BMD test of any kind were assigned the value 1; others were assumed to not have received a test and assigned the value 0.

**Table 1: Key terms relating to SCS:** 

	British Columbia	Ontario

Data Source: Physician Billings	Medical Services Plan Database	Ontario Health Insurance Plan Claims Database

Source Variable	Fee item	Fee code

Description	Fee item	OHIP fee code

Values	08688 — Bone density — Single area	X142 — Bone mineral density — subsequent test — Low risk patient — one site
	08689 — Bone density — Second area	X145 — Bone mineral density — Baseline test — one site
	08696 — Bone density — Whole body	X146 — Bone mineral density — Baseline test — 2+ sites
		X148 — Bone mineral density — Subsequent test — Low risk patient — 2+ sites.
		X149 — Bone mineral density — High risk patient — 1 site
		X152 — Bone mineral density— Low risk — 1 site
		X153 — Bone mineral density — Low risk — 2+ sites
		X155 — Bone mineral density — High risk — 2+ sites

### Calculation

Numerator variable values were summed (giving the total number of people who received a test) and divided by the number of people in the denominator (entire cohort). Proportions are multiplied by 100 to give a percentage. The measure was calculated for 1) overall and 2) sex-specific cohorts. Because we used population data from identically defined cohorts, crude rates were used for comparing performance without standardizing.

## Validation

A key challenge was when one or both provinces had more or better data or better developed methods. To ensure measure comparability across provinces, there was a limit with what could be used (e.g. data or programs such as macros). This created an opportunity for provinces to generate two versions of the indicator: one to compare with other provinces and one to compare to previous provincial work.

### Validation actions taken for our osteoporosis measure:

1. To support validity of provincial comparisons: we used agreed-upon years of data and methods comparable between BC and ON.
2. To assess whether broader inclusion criteria would change results: we added to our cohort everyone aged 65 or older in the first FY, and then used additional longitudinal data (when available) to exclude people who had a BMD test in the three years preceding the study period.
3. To assess validity of within province measurement: for each province, we created the measures using additional data elements (time and/or fee codes) or previously developed methods and compared the results to those from the between-province measure. The more detailed measure: 1) provides a best estimate of within-province performance and 2) and allows estimation of measurement error for the between-province measure.

## Limitations and Generalizability

Defining our cohort based on turning age 65 in the first year of observation avoids capturing people already screened as guideline-recommended testing eligibility begins at this age [2], but this introduces variability such that people are eligible for screening from two to three years depending on their birthdate. Four years of data would remedy this inconsistency; further longitudinal data would allow using a look back approach to exclude those already screened. We could not confirm whether all BMD tests were Dual-energy X-ray Absorptiometry; differentiating between possible tests may refine the accuracy of the concept in the future. Additional considerations for future studies, beyond cohort definition and billing code selection, include BMD testing eligibility and which types of care providers order the referrals.

## Conclusion

We present an overview for developing an administrative data measure to compare osteoporosis screening between BC and ON. Importantly, data could be combined within but not between provinces: our work on developing comparable measures provides a foundation for future projects to make valid inter-provincial performance comparisons [8]—a key stepping stone towards a national level learning health care system [9].

## Computer Code

A sample SAS file used to create the measure in BC is included in Supplementary Appendix 1.

## Supplementary Appendices

Supplementary Appendix 1: contains SAS computer code for operationalizing the Osteoporosis screening measure.

## References

[1] Munce SEP, Allin S, Carlin L, et al. Understanding referral patterns for bone mineral density testing among family physicians: A qualitative descriptive study. J Osteoporos. 1 2016;2016:2937426. Available from: https://www.ncbi.nlm.nih.gov/pmc/articles/PMC4745655/ 10.1155/2016/2937426 PMC4745655 26904357

[2] Papaioannou A, Morin S, Cheung AM, et al. 2010 clinical practice guidelines for the diagnosis and management of osteoporosis in Canada: Summary. CMAJ. 11 2010;182(17):1864–73. 10.1503/cmaj.100771 20940232 PMC2988535

[3] Stukel TA, Croxford R, Rahman F, Bierman A, Galzier R. Variations in Quality Indicators Across Ontario Physician Networks [Internet]. Toronto: Institute for Clinical Evaluative Sciences; 2016 [cited 2019 Jun 26]. Available from: http://proxy.library.carleton.ca/loginurl=https://www.deslibris.ca/ID/10064099

[4] LeMessurier J, O’Donnell S, Walsh P, McRae L, Bancej C. The development of national indicators for the surveillance of osteoporosis in Canada. Chronic Dis Inj Can. 2012;32(2):101–7. 22414307 PMC5104541

[5] Hawker GA, Badley EM, Jaglal S, et al. Musculoskeletal conditions. In: AS B, editor. Project for an Ontario Women’s Health Evidence-Based Report, Vol 2. Toronto; 2010.

[6] Huddle. 2019. Available from: https://www.huddle.com/

[7] Knowlton J, Belnap T, Patelesio B, Priest E, von Recklinghausen F, Taenzer AH. A framework for aligning data from multiple institutions to conduct meaningful analytics. eGEMs. 2017; 5(3):2,1-7. 10.5334/egems.195 PMC5982973 29881753

[8] Canadian Institutes of Health Research. Transforming CBPHC delivery through comprehensive performance measurement and reporting [Internet]. 2013 [cited 2019 Mar 14]. Available from: http://cihr-irsc.gc.ca/e/47163.html

[9] Friedman CP, Allee NJ, Delaney BC, et al. The science of Learning Health Systems: Foundations for a new journal. Learning Health Systems. 1 1 2017;1(1):e10020. 10.1002/lrh2.10020 PMC6516721 31245555

