# Supplementary material for: Achieving cross provincial comparisons of osteoporosis screening performance from administrative health data
Source: Int J Popul Data Sci. 2019 Dec 5;4(1):1116. doi: 10.23889/ijpds.v4i1.1116 (PMC8144816; doi:10.23889/ijpds.v4i1.1116)
Supplement: Supplementary Appendix 1: contains SAS computer code for operationalizing the Osteoporosis screening measure. [file ijpds-04-1116-s001.pdf]

Achieving cross provincial comparisons of osteoporosis screening performance from administrative health data

https://doi.org/10.23889/ijpds.v4i1.1116

Supplementary Appendix 1

```
** Example calculation of Osteoporosis Screening performance measure using
British Columbia data**;

** set the 3 years of physician data, keeping only the fee items for bone
mineral density;
data BMD;
  set phys_ffs_data1314(keep=studyid feeitem where=(feeitem in
('08696','08689','08688')))
      phys_ffs_data1415(keep=studyid feeitem where=(feeitem in
('08696','08689','08688')))
      phys_ffs_data1516(keep=studyid feeitem where=(feeitem in
('08696','08689','08688')));

  keep studyid;
  run;


*keep only 1 record per person to flag that the person had a BMD in the 3-
year period;
proc sort data=BMD nodupkey;
  by studyid;
  run;


** cohortBMD is already limited to the eligible cohort (that is, the
inclusion & exclusion criteria are already applied).;
data cohortBMD;
  merge cohortBMD(in=a) BMD(in=b);
  by studyid;
  if a;

  if a and b then had_BMD=1;
  else if a and not b then had_BMD=0;
  run;

** % who had a BMD can now be found, by region, gender etc as needed;
```
